# Supplementary material for: The genomic epidemiology of Escherichia albertii infecting humans and birds in Great Britain
Source: Nat Commun. 2023 Mar 27;14:1707. doi: 10.1038/s41467-023-37312-3 (PMC10043262; doi:10.1038/s41467-023-37312-3)
Supplement: Supplementary file 1 — Supplementary Information [file 41467_2023_37312_MOESM1_ESM.pdf]

Supplementary information to:

The genomic epidemiology of *Escherichia albertii* infecting humans and birds in Great Britain

Supplementary Methods

Inferred significance of *E. albertii* infection: The inferred significance of *E. albertii* infection to wild and captive zoo bird health (i.e. its likely contribution to the cause of death) was classified as significant, equivocal, or incidental, based on review of the incident history and the pathological, microbiological and parasitological findings for those examined post mortem, as follows:

(A) Significant

Heavy growth (i.e. moderate/predominant/nearly pure/pure growth) of *E. albertii* from the liver and/or small intestinal contents AND no alternative cause of death (COD) identified AND history consistent with infectious disease outbreak (i.e. multiple mortality and/or observed sick birds) OR poor body condition (i.e. thin or emaciated)

(B) Equivocal

1. Heavy growth of *E. albertii* from the liver and/or small intestinal contents (if comment on character of isolate recovery is available) AND alternative infectious COD identified

OR

2. Heavy growth of *E. albertii* from the liver and/or small intestinal contents AND Non-infectious COD identified (i.e. Trauma/Predation/Euthanasia/Other) AND history consistent with infectious disease outbreak (i.e. multiple mortality and/or observed sick birds) OR poor body condition (i.e. thin or emaciated).

(C) Incidental

1. Non-infectious COD identified (i.e. Trauma/Predation/Euthanasia/ Other) AND no history consistent with infectious disease outbreak regardless of character of *E. albertii* growth

OR

2. Scant growth of *E. albertii* only regardless of COD

Bioinformatic detection of LEE pathogenicity island

The presence of LEE island was determined based on percentage of mapping coverage of samples to the *E. albertii* strain 1551-2 reference genome (GenBank accession NZ_CP025317.1) region 4358862-4389747 bp, at a threshold of >70% mapping coverage.

Supplementary Data Titles

Supplementary Data 1 – Details of 83 human *E. albertii* isolates from GB analysed in this study.

Supplementary Data 2 – Details of 79 avian *E. albertii* isolates from GB analysed in this study.

Supplementary Data 3. A representative AMR-containing contiguous sequence identified in 14 *Escherichia albertii* isolates and related sequences.

Supplementary Data 4 – Metadata of 475 global *E. albertii* isolates retrieved from EnteroBase

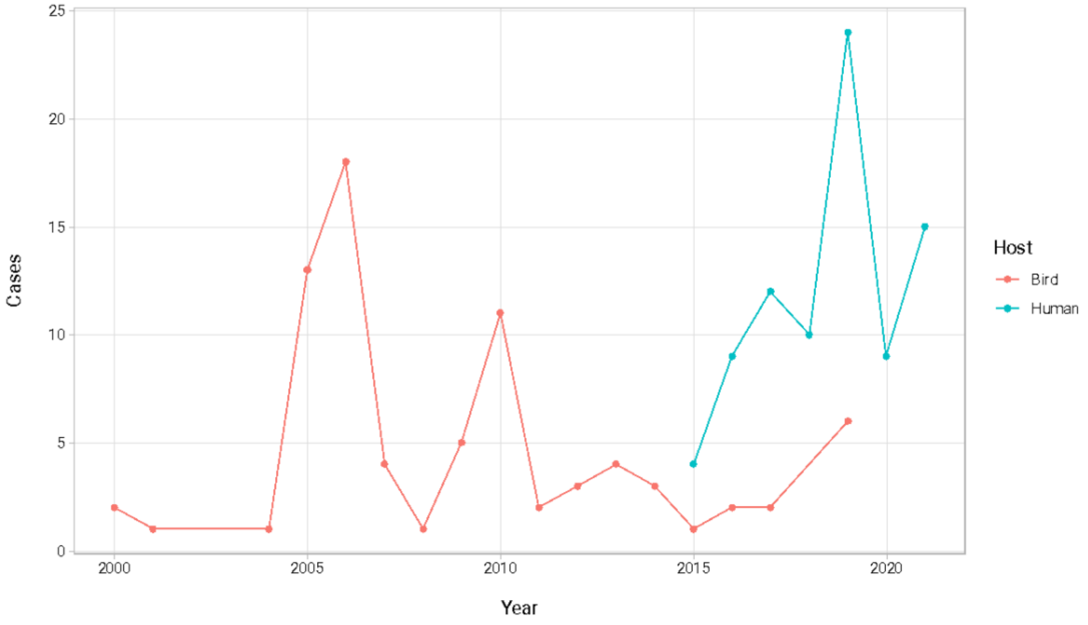

Supplementary Figure 1. Number of human and bird *Escherichia albertii* isolates per year. Human isolates were collected 2015-2021 (blue), while bird isolates were collected 2000-2019 inclusive (pink).

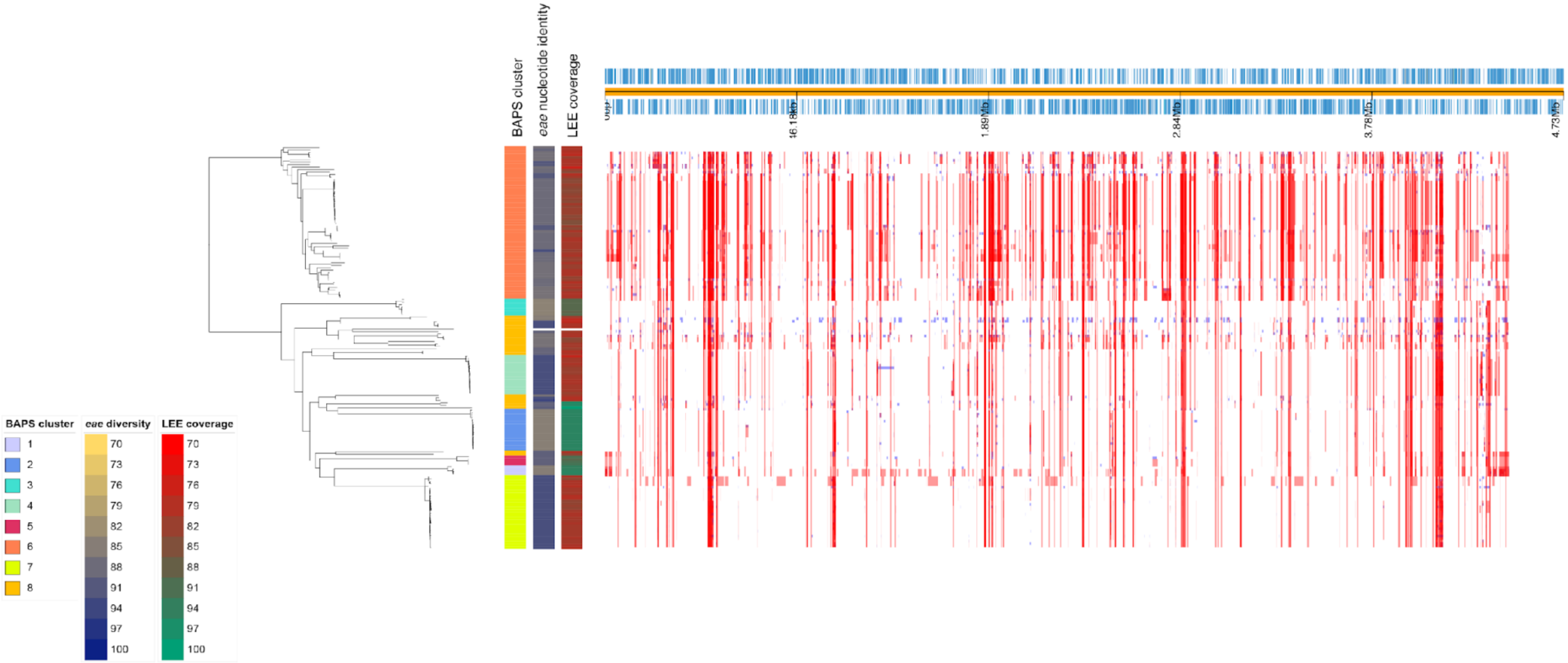

Supplementary Figure 2. Detection of putative regions of recombination and virulence determinants across *E. albertii* genomes. Midpoint rooted maximum likelihood tree of 162 *E. albertii* isolates is displayed on the left. The three columns in the centre of the figure show the BAPS clusters, percentage of eae nucleotide diversity and percentage of mapping coverage across the LEE pathogenicity island, from left to right and coloured according to the inlaid keys. The areas of putative recombination (gubbins outputs) are shown on the right with red coloured blocks highlighting regions of putative recombination shared across multiple isolates through common descent and blue coloured blocks being unique to a single isolate. The yellow bar above shows the genome position of *E. albertii* strain 1551-2 reference genome (GenBank accession CP025317) with blue blocks showing gene features on the forward (upper) and reverse (lower) strands
