# Supplementary material for: The genomic epidemiology of Escherichia albertii infecting humans and birds in Great Britain
Source: Nat Commun. 2023 Mar 27;14:1707. doi: 10.1038/s41467-023-37312-3 (PMC10043262; doi:10.1038/s41467-023-37312-3)
Supplement: Supplementary file 3 — Description of Additional Supplementary Files [file 41467_2023_37312_MOESM3_ESM.pdf]

Description of Additional Supplementary Files

File Name: Supplementary Data 1
Description: Details of 83 human *E. albertii* isolates from GB analysed in this study

File Name: Supplementary Data 2
Description: Details of 79 avian *E. albertii* isolates from GB analysed in this study.

File Name: Supplementary Data 3
Description: A representative AMR-containing contiguous sequence identified in 14 *Escherichia albertii* isolates and related sequences.

File Name: Supplementary Data 4
Description: Metadata of 475 global *E. albertii* isolates retrieved from EnteroBase
