# Supplementary material for: The genomic epidemiology of Escherichia albertii infecting humans and birds in Great Britain
Source: Nat Commun. 2023 Mar 27;14:1707. doi: 10.1038/s41467-023-37312-3 (PMC10043262; doi:10.1038/s41467-023-37312-3)
Supplement: Supplementary file 5 — Reporting Summary [file 41467_2023_37312_MOESM5_ESM.pdf]

nature portfolio

Corresponding author(s): Kate Baker

Last updated by author(s): Mar 3, 2023

Reporting Summary

Nature Portfolio wishes to improve the reproducibility of the work that we publish. This form provides structure for consistency and transparency in reporting. For further information on Nature Portfolio policies, see our Editorial Policies and the Editorial Policy Checklist.

Statistics

For all statistical analyses, confirm that the following items are present in the figure legend, table legend, main text, or Methods section.

n/a	Confirmed	
☐	☒	The exact sample size (*n*) for each experimental group/condition, given as a discrete number and unit of measurement
☐	☒	A statement on whether measurements were taken from distinct samples or whether the same sample was measured repeatedly
☐	☒	The statistical test(s) used AND whether they are one- or two-sided<br>*Only common tests should be described solely by name; describe more complex techniques in the Methods section.*
☐	☒	A description of all covariates tested
☒	☐	A description of any assumptions or corrections, such as tests of normality and adjustment for multiple comparisons
☐	☒	A full description of the statistical parameters including central tendency (e.g. means) or other basic estimates (e.g. regression coefficient) AND variation (e.g. standard deviation) or associated estimates of uncertainty (e.g. confidence intervals)
☐	☒	For null hypothesis testing, the test statistic (e.g. *F*, *t*, *r*) with confidence intervals, effect sizes, degrees of freedom and *P* value noted<br>*Give P values as exact values whenever suitable.*
☒	☐	For Bayesian analysis, information on the choice of priors and Markov chain Monte Carlo settings
☒	☐	For hierarchical and complex designs, identification of the appropriate level for tests and full reporting of outcomes
☒	☐	Estimates of effect sizes (e.g. Cohen's *d*, Pearson's *r*), indicating how they were calculated

Our web collection on statistics for biologists contains articles on many of the points above.

Software and code

Policy information about availability of computer code

Data collection	Data was collected during routine surveillance and stored in UKHSA and ZSL databases, and extracted in Microsoft Excel for analysis in this study.
Data analysis	The following software packages were used: Trimmomatic v0.38, FastQC v0.11.6, MultiQC v1.7, BWA mem v0.7.17, SAMtools suite v1.9-47, Picard v2.21.1-SNAPSHOT MarkDuplicates, BCFtools suite v1.9-80, BEDTools v2.28.0 maskfasta, Gubbins v2.3.4, SNP-sites v2.4.1, IQ-TREE v2.0-rc2, iTOL v6.5, RhierBAPS v1.1.3, Unicycler v0.4.7, QUAST v5.0.2, Prokka v1.13.3, AMRFinderPlus v3.9.3, UpSetR v2.1.3.

For manuscripts utilizing custom algorithms or software that are central to the research but not yet described in published literature, software must be made available to editors and reviewers. We strongly encourage code deposition in a community repository (e.g. GitHub). See the Nature Portfolio guidelines for submitting code & software for further information.

Data

Policy information about availability of data

All manuscripts must include a data availability statement. This statement should provide the following information, where applicable:

- Accession codes, unique identifiers, or web links for publicly available datasets
- A description of any restrictions on data availability
- For clinical datasets or third party data, please ensure that the statement adheres to our policy

Individual accessions numbers for isolates used in this study area available in Supplementary Data 1, 2, and 4. Phylogenetic trees from this study have been

deposited in FigShare (DOI: 10.6084/m9.figshare.20894854.v1). The authors recognise that this study opens up important further avenues for functional research of E. albertii and are happy to make isolates available on request.

Human research participants

Policy information about studies involving human research participants and Sex and Gender in Research.

Reporting on sex and gender	NA
Population characteristics	NA
Recruitment	NA
Ethics oversight	NA

Note that full information on the approval of the study protocol must also be provided in the manuscript.

Field-specific reporting

Please select the one below that is the best fit for your research. If you are not sure, read the appropriate sections before making your selection.

☒ Life sciences ☐ Behavioural & social sciences ☐ Ecological, evolutionary & environmental sciences

For a reference copy of the document with all sections, see nature.com/documents/nr-reporting-summary-flat.pdf

Life sciences study design

All studies must disclose on these points even when the disclosure is negative.

Sample size	Sample size was not calculated as this represents discrete and complete surveillance collections.
Data exclusions	No data were excluded from the study.
Replication	100 bootstrap replicates of maximum likelihood phylogenetic trees were conducted and are displayed on the figures. Low replicability of some branch groupings are attributable to poorly defined population structure in the pathogen and are discussed in the results and have been resolved during the peer review process.
Randomization	The sampling frame for both bird and human isolates was comprehensive to there was no need to randomise.
Blinding	The data analyst involved in study did not link metadata to genome data until phylogenetic trees had been generated so was blind to the origin of samples until their relationships were known. Also, the authors did not have any pre-supposed hypotheses about the relationships among human and bird isolates so blinding was not unnecessary in this case.

Reporting for specific materials, systems and methods

We require information from authors about some types of materials, experimental systems and methods used in many studies. Here, indicate whether each material, system or method listed is relevant to your study. If you are not sure if a list item applies to your research, read the appropriate section before selecting a response.

Materials & experimental systems

n/a	Involved in the study	
☒	☐	Antibodies
☒	☐	Eukaryotic cell lines
☒	☐	Palaeontology and archaeology
☒	☐	Animals and other organisms
☒	☐	Clinical data
☒	☐	Dual use research of concern

Methods

n/a	Involved in the study	
☒	☐	ChIP-seq
☒	☐	Flow cytometry
☒	☐	MRI-based neuroimaging
